# Urinary Stone Recurrence After Initiation of GLP-1-Based Therapy: A Romanian Retrospective Cohort Study of Clinical, Renal, and Hydration-Related Factors

**DOI:** 10.3390/jcm15208039

**Published:** 2026-10-16

**Authors:** Razvan Alexandru Danau, Madalina Andreea Munteanu, Andrei Mitel, Gabriel Predoiu, Ileana Secareanu, Viorel Jinga

**Affiliations:** 1 Department of Urology, “Carol Davila” University of Medicine and Pharmacy, 8 Eroii Sanitari Blvd., 050474 Bucharest, Romania; razvan.danau@umfcd.ro (R.A.D.); mitel_andrei@yahoo.com (A.M.); gabriel.predoiu@umfcd.ro (G.P.); viorel.jinga@umfcd.ro (V.J.); 2 Department of Urology, “Prof. Dr. Th. Burghele” Clinical Hospital, 20 Panduri Str., 050659 Bucharest, Romania; 3 Department of Cardiology, “Carol Davila” University of Medicine and Pharmacy, 8 Eroii Sanitari Blvd., 050474 Bucharest, Romania; 4 Diabetology Department, Rosana Medical Center, 062079 Bucharest, Romania; ileana_secareanu@yahoo.com; 5 Medical Sciences Section, Academy of Romanian Scientists, 050085 Bucharest, Romania

**Keywords:** GLP-1 receptor agonist, semaglutide, tirzepatide, nephrolithiasis, urinary stone disease, obesity, diabetes mellitus

## Abstract

**Background:** Glucagon-like peptide-1 receptor agonists and dual GIP/GLP-1 receptor agonists produce substantial weight loss and improve glycemic control, but their relationship with urinary stone recurrence remains incompletely characterized. We evaluated clinically and radiologically confirmed recurrent stone episodes occurring after initiation of semaglutide or tirzepatide. **Methods:** This retrospective single-center cohort screened 168 adults receiving semaglutide or tirzepatide. Four patients with previous bariatric surgery, 33 without at least 6 months of post-initiation clinical follow-up, and five with hyperparathyroidism were excluded, leaving 126 participants. Follow-up was calculated from GLP-1-based therapy initiation to the last documented clinical or imaging assessment. Recurrence required a stone episode documented before treatment initiation and a new symptomatic or radiologically progressive stone event after initiation. **Results:** The cohort included 94 semaglutide and 32 tirzepatide users. Median follow-up was 9 months [IQR 8–12]. Thirteen patients (10.3%; 95% CI 6.1–16.9%) had a recurrent episode after treatment initiation. No measured demographic, metabolic, renal, hydration-related, electrolyte, or treatment characteristic clearly differentiated patients with and without recurrence. Allopurinol use was more frequent among recurrent stone formers in an unadjusted analysis, a finding considered exploratory and likely affected by confounding by indication. **Conclusions:** In this single-arm cohort, 10.3% of patients had clinically and radiologically documented stone recurrence after GLP-1-based therapy initiation. The study describes post-initiation events but cannot estimate treatment-attributable risk because it lacked an untreated comparator, pre-treatment incidence-rate analysis, standardized surveillance, and complete exposure data. Larger prospective comparator studies with baseline imaging, time-to-event follow-up, complete urinary chemistry, and treatment-dose data are required.

## 1. Introduction

Urinary stone disease is a common and frequently recurrent condition determined by interactions among genetic susceptibility, diet, fluid intake, metabolic disease, medication exposure, and urinary anatomy. Obesity and type 2 diabetes mellitus are particularly relevant because both are associated with nephrolithiasis and with adverse urinary profiles, including lower urine pH, altered ammonium excretion, hyperuricosuria, and increased risk of uric acid stones [1,2,3]. As the prevalence of obesity and diabetes increases, urologists increasingly encounter patients in whom stone prevention must be integrated with modern metabolic and weight-management therapy.

Glucagon-like peptide-1 receptor agonists, including semaglutide, and dual glucose-dependent insulinotropic polypeptide/GLP-1 receptor agonists, including tirzepatide, produce clinically meaningful reductions in body weight and improve glycemic control. These changes could theoretically reduce stone risk by improving insulin resistance, reducing dietary intake, and modifying obesity-related urinary abnormalities. Conversely, gastrointestinal adverse effects such as nausea, vomiting, and reduced oral intake may cause transient dehydration, lower urine volume, and increased urinary supersaturation, particularly during initiation and dose escalation. The net effect of GLP-1-based therapy on urinary stone formation is therefore uncertain and may differ according to baseline metabolic phenotype, hydration behavior, kidney function, and concomitant medication use.

The principal study motivating the present investigation was conducted by Feghali and colleagues [4]. In that retrospective cohort, obese stone-forming patients who lost weight while receiving GLP-1-based therapy underwent paired 24 h urine collections before and after treatment. The investigators reported substantial weight loss without significant adverse changes in urine calcium, citrate, uric acid, sodium, or other major lithogenic parameters. Urine oxalate decreased significantly, and the authors concluded that GLP-1-based weight loss did not appear to worsen stone-forming risk based on available urine chemistry. That study provided important metabolic reassurance, but it focused on previously established stone formers, used urine chemistry rather than clinically adjudicated incident events as its main framework, and did not resolve whether GLP-1-based therapy modifies real-world stone recurrence.

Subsequent analyses have added complexity. Another study compared 24 h urine parameters among patients with diabetes receiving SGLT2 inhibitors, GLP-1 receptor agonists, or neither treatment and found that SGLT2 inhibition was associated with several urinary changes, whereas GLP-1 receptor agonist exposure showed few independent associations with urine analytes [5]. Large comparative pharmacoepidemiologic studies have reported lower nephrolithiasis rates among SGLT2 inhibitor initiators than among GLP-1 receptor agonist initiators [6,7]. However, those comparisons do not demonstrate that GLP-1 receptor agonists increase risk relative to no treatment; they may instead reflect a protective effect of SGLT2-induced osmotic diuresis, differences in prescribing, or residual confounding.

Concomitant treatments further complicate interpretation. Metformin may improve glycemic control without consistently changing 24 h urinary stone parameters [8]. Allopurinol is an established treatment for selected patients with hyperuricosuria and recurrent calcium oxalate stones [9]. Thiazide and thiazide-like diuretics have traditionally been used to reduce calciuria, although recent randomized evidence has questioned the magnitude of their recurrence-prevention benefit and highlighted dose-related adverse effects [10]. SGLT2 inhibitors may affect urine volume, citrate, pH, and uric acid handling [5,6,7]. Consequently, medication exposure should be described when evaluating stone disease in patients receiving GLP-1-based therapies.

Baseline electrolyte and pharmacotherapy profiles may help distinguish direct treatment effects from the metabolic context in which GLP-1-based agents are prescribed. Serum sodium and potassium primarily reflect systemic homeostasis rather than urinary supersaturation, but they provide clinically relevant information regarding hydration, gastrointestinal losses, renal reserve, and exposure to diuretics or other cardio–renal therapies. At the same time, metformin, SGLT2 inhibitors, allopurinol, and diuretics may alter glycemic status, osmotic diuresis, uric acid metabolism, urinary calcium, or electrolyte balance. Evaluating these variables at treatment initiation is therefore important for phenotyping the cohort and for identifying potential confounding by co-treatment.

The present study aimed to characterize the prevalence of clinically and radiologically documented recurrent urinary stone disease in a Romanian cohort receiving semaglutide or tirzepatide. Secondary objectives were to compare demographic, metabolic, renal, hydration-related, and GLP-1 treatment characteristics between participants with and without recurrent stone disease and to describe concomitant use of metformin, SGLT2 inhibitors, allopurinol, and diuretics. We hypothesized that recurrent stone formers might demonstrate lower urine volume, greater weight loss, poorer glycemic control, or reduced kidney function; however, the study was explicitly designed as exploratory because no untreated comparator was available.

## 2. Materials and Methods

### 2.1. Study Design and Population

**Figure 1:**
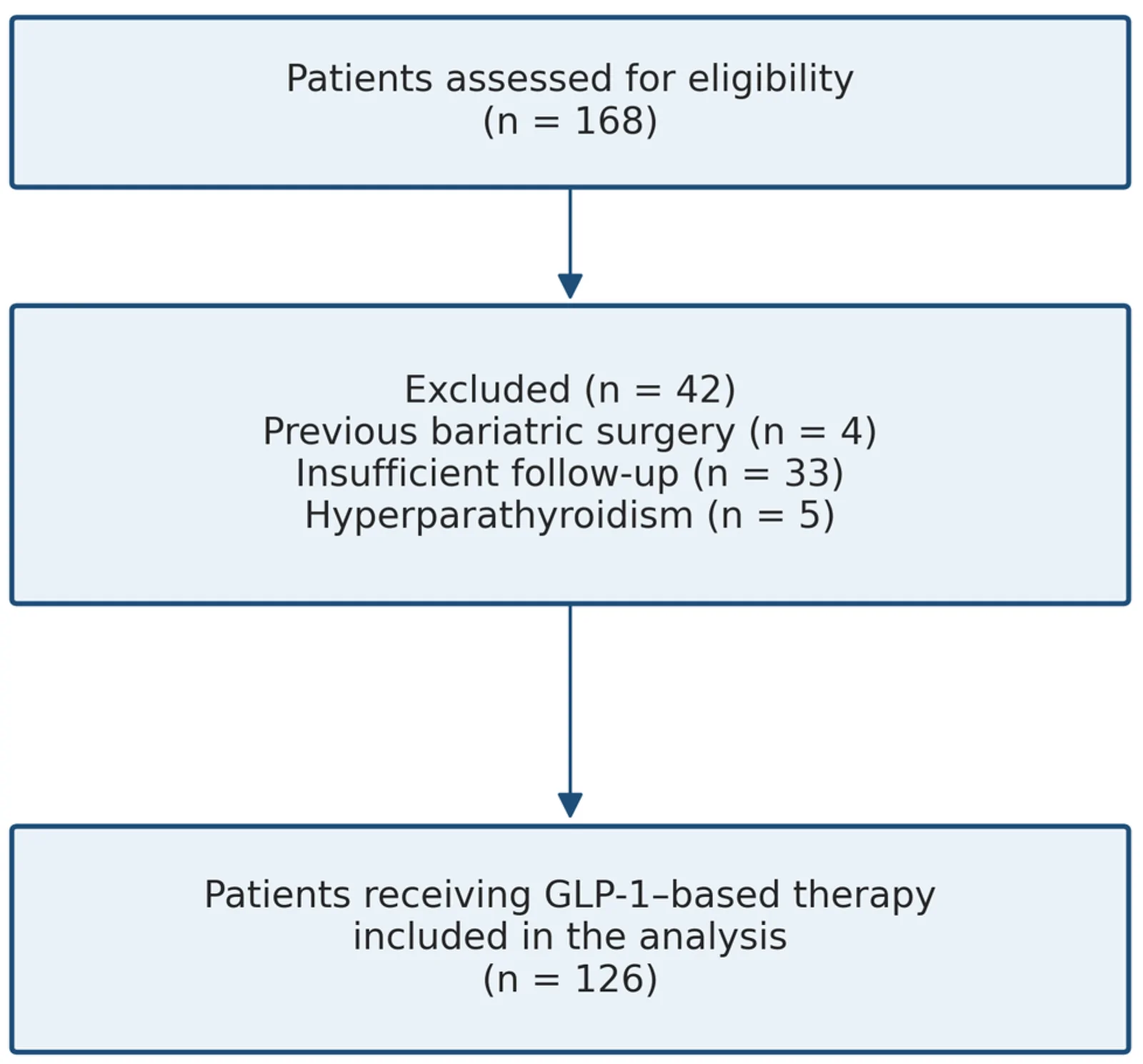
Flow diagram of study screening, exclusions, and final cohort selection.

We performed a retrospective observational single-center cohort study of adults receiving GLP-1-based therapy at Clinical Hospital “Prof. Dr. Th. Burghele”, Bucharest, Romania, between 2024 and 2026. A total of 168 potentially eligible patients were screened. We excluded four patients with previous bariatric surgery because bariatric procedures can substantially modify intestinal oxalate absorption and stone risk, 33 patients who did not have at least 6 months of documented clinical follow-up after GLP-1-based therapy initiation, and five patients with hyperparathyroidism because this represents an independent cause of hypercalcemia, hypercalciuria, and nephrolithiasis as we can see in Figure 1. The final analytical cohort comprised 126 patients. Follow-up was calculated from the recorded date of semaglutide or tirzepatide initiation to the date of the last available urological, metabolic, or imaging assessment. The six-month threshold was selected a priori to exclude patients with insufficient opportunity for post-initiation outcome assessment; however, this restriction may have introduced selection bias and does not ensure equal observation time. The study was conducted in accordance with the Declaration of Helsinki and was approved by the Institutional Ethics Committee of Clinical Hospital “Prof. Dr. Th. Burghele”, Bucharest, Romania (7058/21 July 2025). The requirement for individual informed consent was waived because the analysis was retrospective and used pseudonymized clinical data.

### 2.2. Exposure and Clinical Variables

GLP-1-based treatment was categorized as semaglutide or tirzepatide. The index date was the documented date of treatment initiation. Available variables included treatment indication, reimbursement status, age, sex, smoking, dyslipidemia, baseline BMI, most recent BMI, weight change, diabetes status, most recent HbA1c, serum creatinine, eGFR, documented gastrointestinal adverse effects, and follow-up duration. Weight change was expressed in kilograms, with negative values indicating weight loss. Baseline serum sodium and potassium at treatment initiation were prespecified as systemic electrolyte variables. Exact dose-escalation schedules, adherence, interruptions, and cumulative exposure were not consistently recoverable from the records and therefore were not analyzed. These missing exposure details limit dose-response and agent-specific interpretation.

Concomitant medications of potential relevance to urinary stone disease included metformin, SGLT2 inhibitors (empagliflozin or dapagliflozin), allopurinol, and chronic diuretic therapy. Metformin was generally prescribed at 1 g twice daily, and allopurinol at 100 mg/day. Diuretic exposure included thiazide, thiazide-like, and loop diuretics; because drug-specific exposure was not consistently available, these agents were analyzed as a combined category. Patients received routine counseling regarding a balanced calorie-restricted diet, avoidance of sugar-containing beverages, portion control, and aerobic and resistance exercise. Gastrointestinal adverse effects were abstracted when explicitly documented, but retrospective recording was incomplete and did not permit reliable comparison by recurrence status; this variable was therefore not used for inferential analysis.

### 2.3. Stone Assessment and Outcomes

Urinary stone disease was assessed by review of the urological clinical record together with available imaging. The exposure index date was initiation of semaglutide or tirzepatide. For the primary outcome, recurrence required: (1) a clinically or radiologically documented stone episode before GLP-1-based therapy initiation and (2) a subsequent stone event after initiation that was clinically compatible and radiologically confirmed. Thus, all 13 outcome events occurred after initiation of GLP-1-based therapy. Available imaging comprised ultrasonography and/or non-contrast computed tomography performed according to routine care rather than a fixed research schedule. A post-initiation event was classified as recurrence when imaging demonstrated a new calculus, migration associated with a clinical episode, or documented interval growth/progression judged by the treating urologist; unchanged persistence of a known calculus without a new clinical episode was not counted as recurrence. Because imaging was symptom-driven or clinician-directed rather than protocolized, ascertainment intensity may have differed among patients. The comparison group included patients without a post-initiation recurrent event and could therefore include both patients with no prior stone history and patients with a single pre-treatment episode. The available aggregate dataset did not permit a reliable subgroup analysis restricted to baseline stone formers. The primary analysis consequently estimates the proportion with post-initiation recurrence within the treated cohort rather than incidence attributable to therapy. A 24 h urine volume was available for 125 patients. Collection timing was not uniformly standardized relative to treatment initiation or the recurrent event; urine volume was therefore interpreted as a descriptive hydration-related measure rather than a causal predictor.

### 2.4. Statistics

Continuous variables were assessed for completeness and distributional characteristics and summarized using median and interquartile range, with mean and standard deviation additionally reported for overall description. Between-group comparisons used the Mann–Whitney U test because only 13 patients met the recurrent-stone outcome. Categorical variables were summarized as counts and percentages and compared using Fisher’s exact test. Unadjusted odds ratios with 95% confidence intervals were calculated from 2 × 2 contingency tables. The 95% confidence interval for the proportion with post-initiation recurrence was calculated using the Wilson method. Sodium and potassium were analyzed as continuous baseline variables using complete cases. A multivariable model was not fitted because 13 events were insufficient for stable simultaneous adjustment of medication exposures and relevant clinical covariates. All analyses were exploratory. Statistical non-significance was not interpreted as evidence of equivalence or absence of association, and effect estimates and confidence intervals were prioritized over *p* values.

## 3. Results

### 3.1. Study Population and GLP-1-Based Therapy

Of 168 screened patients, 42 were excluded and 126 were included. Median age was 67.0 years [IQR 62.25–70.00], and 59 patients (46.8%) were female. Dyslipidemia was present in 112 (88.9%), smoking was recorded in 42 (33.3%), and diabetes mellitus was present in 103 (81.7%). Median baseline BMI was 32.0 kg/m^2^ [30.25–34.00]. The median documented weight change was −15.0 kg [−17.25 to −14.00], indicating substantial weight loss during the observed treatment period. Median HbA1c was 6.9% [6.7–7.0] as we can see in Table 1.

Semaglutide was used by 94 patients (74.6%) and tirzepatide by 32 (25.4%). Median post-initiation follow-up was 9 months [IQR 8–12]; all included patients had at least 6 months of documented follow-up. Although the treatment start date was available to define the index date and establish that recurrence occurred after initiation, exact dose, titration, adherence, interruptions, and cumulative exposure were incompletely documented and were not used in comparative inference. Median serum creatinine was 1.00 mg/dL [0.90–1.00], median eGFR was 69.5 mL/min/1.73 m^2^ [60.0–77.0], and median 24 h urine volume was 2200 mL [1990–2350]. The timing of urine collection relative to recurrence was not uniform.

### 3.2. Concomitant Medications

Metformin was used by 80 patients (63.5%), SGLT2 inhibitors by 32 (25.4%), allopurinol 100 mg/day by 52 (41.3%), and diuretics by 31 (24.6%). Metformin exposure was similar among patients with and without recurrent stones (61.5% vs. 63.7%; OR 0.91, 95% CI 0.28–2.97; *p* = 1.000). SGLT2 inhibitor use was numerically less frequent among recurrent stone formers (15.4% vs. 26.5%; OR 0.50, 95% CI 0.11–2.40; *p* = 0.513), as was diuretic use (15.4% vs. 25.7%; OR 0.53, 95% CI 0.11–2.52; *p* = 0.517). Allopurinol was more frequent among recurrent stone formers (69.2% vs. 38.1%; OR 3.66, 95% CI 1.06–12.63; *p* = 0.039) as we can observe in Table 2. Given the retrospective design, limited event count, and likely prescription of allopurinol for hyperuricemia, gout, or prior stone risk, this association was considered susceptible to confounding by indication and reverse causation.

**Figure 2:**
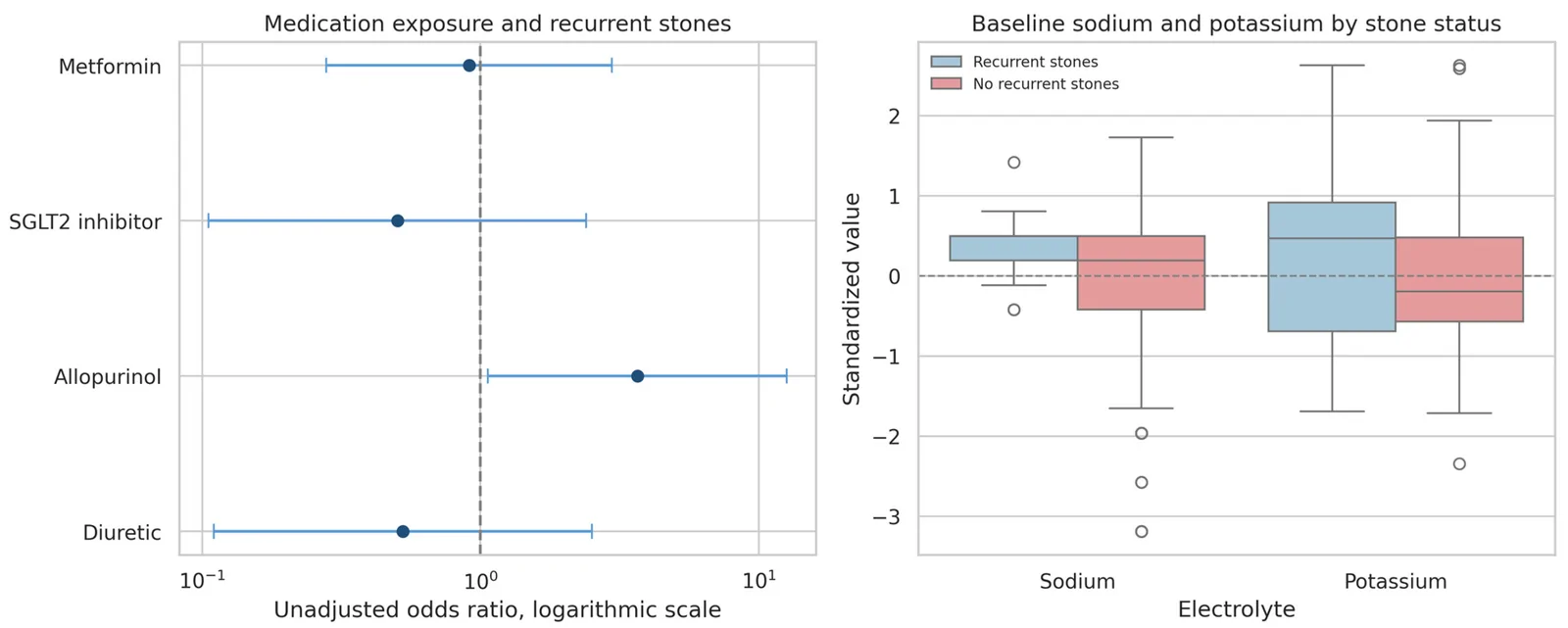
Unadjusted medication associations and standardized baseline electrolyte distributions according to recurrent stone status. Odds ratios are displayed on a logarithmic scale.

Baseline sodium was available for 125 patients and had a median of 139.0 mmol/L [137.0–140.0] as we can see in Table 3. Median sodium was 140.0 mmol/L among recurrent stone formers and 139.0 mmol/L among patients without recurrent stones (*p* = 0.186). Baseline potassium was also available for 125 patients, with a median of 4.31 mmol/L [4.12–4.65]. Potassium did not differ between recurrent stone formers and patients without recurrent disease (4.63 vs. 4.30 mmol/L; *p* = 0.692) as we can observe in Figure 2.

### 3.3. Recurrent Urinary Stone Disease

Thirteen of 126 patients had recurrent urinary stone disease after initiation of GLP-1-based therapy, confirmed through clinical and radiological assessment, corresponding to 10.3% (95% CI 6.1–16.9%) of the treated cohort. Each of these patients had a documented pre-treatment stone episode followed by a confirmed post-initiation event. The available aggregate dataset did not retain sufficiently complete event dates to report a reliable patient-level median time from initiation to recurrence; therefore, no time-to-event estimate was calculated. Recurrent disease was identified in 11 of 94 semaglutide users (11.7%) and two of 32 tirzepatide users (6.3%); the between-agent comparison was not statistically significant (*p* = 0.513) and was underpowered.

Median age was identical at 67 years in patients with and without recurrent stones. Recurrent stone formers had a median BMI of 33.0 versus 32.0 kg/m^2^ and median weight change of −14.5 versus −16.0 kg, but neither difference was significant. Median 24 h urine volume was 2300 mL among recurrent stone formers and 2200 mL among patients without recurrent disease (*p* = 0.804). Median serum creatinine was 1.00 mg/dL in both groups, while median eGFR was numerically lower among recurrent stone formers at 64 versus 70 mL/min/1.73 m^2^, without statistical significance (*p* = 0.379). We can observe all of these in Table 4

Diabetes was present in 84.6% of recurrent stone formers and 81.4% of patients without recurrent stones (*p* = 1.000). No significant associations were observed for sex, smoking, dyslipidemia, HbA1c, or follow-up duration. These null comparisons should be interpreted in the context of only 13 recurrent-stone events and limited statistical power.

## 4. Discussion

### 4.1. Principal Findings

This study addresses a clinically relevant but underexplored question: the occurrence of recurrent urinary stone events after initiation of GLP-1-based therapy in routine practice. Its principal contribution is the clinical and radiological documentation of post-initiation recurrence in a Romanian cohort, while its design does not permit attribution of these events to the medication itself.

Among 126 adults receiving semaglutide or tirzepatide, 13 (10.3%) experienced a clinically and radiologically confirmed recurrent stone event after treatment initiation. No measured demographic, metabolic, renal, hydration-related, electrolyte, or treatment characteristic clearly differentiated patients with and without recurrence. These null comparisons are imprecise because only 13 events occurred and should not be interpreted as evidence that the assessed variables are unrelated to recurrence. The apparently adequate 24 h urine volume in both groups is descriptive, particularly because collection timing was not standardized relative to the outcome.

The findings should be interpreted as descriptive and hypothesis-generating. Establishing that the recurrent episodes occurred after treatment initiation strengthens temporal characterization, but temporality alone does not establish causality. Every participant received GLP-1-based therapy, and the study lacked an untreated or active-comparator group, a pre-treatment incidence-rate period, standardized imaging, and complete dose-exposure data. Accordingly, the observed 10.3% is the proportion of this selected treated cohort with a post-initiation recurrence during a median 9 months of follow-up, not an estimate of excess risk caused by semaglutide or tirzepatide.

### 4.2. Comparison with the Foundational 24-Hour Urine Study

The study by Feghali et al. [4] was the conceptual foundation for our investigation. Their cohort included obese patients with established nephrolithiasis who underwent paired 24 h urine collections before and after weight loss with GLP-1-based therapy. Median weight loss was substantial, but urine calcium, citrate, uric acid, sodium, volume, and supersaturation indices did not change adversely; urine oxalate decreased. These findings suggested that pharmacological weight loss with GLP-1-based therapy may differ metabolically from malabsorptive bariatric surgery, in which enteric hyperoxaluria and increased calcium oxalate stone risk are well recognized. Our study extends that metabolic framework in three ways. First, we evaluated a Romanian population receiving semaglutide or tirzepatide in routine practice. Second, the outcome was based on clinically and radiologically documented recurrent stone disease rather than urine chemistry alone. Third, we considered renal function, hydration as measured by 24 h urine volume, diabetes, glycemic control, and several relevant concomitant medication classes. However, our dataset lacked sufficiently complete urine calcium, oxalate, citrate, uric acid, sodium, pH, and supersaturation values. The two studies are therefore complementary rather than directly comparable: Feghali et al. provide paired mechanistic evidence, whereas our study provides cross-sectional clinical characterization.

The median weight reduction in our cohort was approximately 15 kg, broadly consistent with clinically meaningful treatment response. We did not observe greater weight loss among recurrent stone formers. This is reassuring but does not establish safety because weight-loss velocity, dietary composition, lean-mass change, gastrointestinal symptoms, and treatment titration were not fully captured. Rapid weight reduction might influence fluid and dietary intake even when the final magnitude of weight loss is similar.

### 4.3. GLP-1 Therapy, Hydration, and Urinary Chemistry

Several opposing mechanisms could connect GLP-1-based therapy with stone risk. Improved insulin sensitivity and glycemic control may ameliorate the low urinary pH characteristic of type 2 diabetes and reduce factors promoting uric acid crystallization [10,11,12]. Weight loss may also improve blood pressure, dietary quality, and metabolic syndrome. Conversely, nausea, vomiting, delayed gastric emptying, early satiety, and reduced thirst or oral intake may cause episodic dehydration. Such short periods may be clinically relevant even when a later 24 h collection shows an apparently adequate volume.

Median 24 h urine volume exceeded 2 L in both groups, and no statistically significant difference was detected. However, collections were obtained during routine care and were not uniformly timed before recurrence. A collection obtained after a stone episode may reflect intensified fluid intake following counseling and cannot be assumed to represent pre-event exposure. Urine volume is therefore reported as a descriptive hydration-related measure rather than a comparative risk factor. Baseline sodium and potassium also did not differ significantly by recurrence status, but single serum electrolyte measurements cannot capture episodic dehydration during nausea, vomiting, reduced oral intake, or dose escalation.

Another study found few independent associations between GLP-1 receptor agonist use and 24 h urine analytes after adjustment, while SGLT2 inhibitors demonstrated more distinct effects [5,13]. These results reinforce the possibility that GLP-1 therapy is metabolically neutral with respect to major urinary stone parameters. However, neutral average chemistry does not exclude clinically important effects in subgroups with pre-existing stones, chronic kidney disease, severe gastrointestinal adverse effects, or inadequate fluid intake.

### 4.4. Concomitant Medication and Stone Risk

Metformin was the most common concomitant therapy, used by 63.5% of participants, and showed no association with recurrent stone disease. Although metformin improves insulin sensitivity and glycemic control, published data in diabetic stone formers have not demonstrated consistently favorable 24 h urine profiles. The present null association is therefore biologically plausible but remains imprecise because treatment duration, dose, adherence, and pre-treatment urinary chemistry were unavailable.

SGLT2 inhibitors were used by 25.4% of participants and were numerically less frequent among recurrent stone formers, although the confidence interval was wide and included both benefit and harm. SGLT2 inhibitors induce glycosuria and osmotic diuresis and can influence urine volume, citrate, pH, uric acid, calcium, magnesium, and phosphate handling. Large observational comparisons have reported lower nephrolithiasis rates among SGLT2 inhibitor initiators than among GLP-1 receptor agonist initiators [12,13,14]. Our point estimate was directionally consistent with a potentially lower prevalence, but the study was not powered or designed to establish a protective effect.

Allopurinol 100 mg/day was used by 41.3% of the cohort and was significantly more frequent among recurrent stone formers in the unadjusted analysis. This finding should not be interpreted as evidence that allopurinol causes stones. Allopurinol is commonly initiated in patients with hyperuricemia, gout, hyperuricosuria, uric acid stones, or recurrent calcium oxalate stones associated with excessive urinary uric acid. The observed association is therefore highly compatible with confounding by indication: patients at greater underlying stone risk were more likely to receive allopurinol. The dose of 100 mg/day may also represent renal-dose adjustment or treatment initiation rather than the dose required to normalize urinary uric acid. Serum uric acid, urinary uric acid, stone composition, treatment start date, and therapeutic response were unavailable, preventing assessment of treatment effectiveness [9,10,11,12,13,14].

Diuretics were used by 24.6% of patients and were not associated with recurrent stone disease. Interpretation is limited because thiazide or thiazide-like agents and loop diuretics were grouped together despite opposing effects on urinary calcium. Thiazides can reduce urinary calcium, whereas loop diuretics may increase calciuria. Indication also matters, because diuretic users may have hypertension, heart failure, edema, or chronic kidney disease, each of which can influence hydration and renal handling. Drug-specific exposure, dose, duration, blood pressure, urinary calcium, and potassium should be incorporated into future analyses.

Baseline sodium and potassium were available for 125 patients and were not associated with recurrent stone status. Serum sodium is tightly regulated and should not be treated as a surrogate for dietary sodium intake or 24 h urinary sodium, which is more directly relevant to calciuria. Serum potassium is likewise distinct from urinary potassium and citrate, but it remains clinically important in patients receiving diuretics, SGLT2 inhibitors, renin–angiotensin system blockers, or potassium citrate. The absence of an observed difference reduces concern regarding major baseline electrolyte imbalance but does not exclude transient abnormalities during gastrointestinal adverse effects or medication titration.

### 4.5. Semaglutide Versus Tirzepatide

Recurrent stones were numerically more frequent among semaglutide users than tirzepatide users, but this comparison was not statistically significant and should not be interpreted as equivalence or as evidence of an agent-specific effect. Semaglutide users comprised approximately three quarters of the cohort, only two events occurred among tirzepatide users, and detailed dose and exposure duration were unavailable. Comparative assessment requires balanced treatment groups, baseline imaging, complete dose and duration data, and adequately powered time-to-event methods.

### 4.6. Clinical Implications

The present data do not support avoiding GLP-1-based therapy solely because of a stone history, but they also cannot establish stone safety or benefit. Clinicians should document baseline stone history, distinguish persistent stones from new or progressive events, encourage guideline-concordant fluid intake, and actively assess nausea, vomiting, and reduced oral intake during treatment initiation and escalation. Patients with recurrent stones warrant stone analysis when available, complete metabolic assessment, and follow-up imaging selected according to symptoms, stone characteristics, and recurrence risk.

The study also demonstrates why clinically confirmed events and urine chemistry should be evaluated together. A patient can have apparently adequate urine volume and still form stones because of hypercalciuria, hypocitraturia, hyperoxaluria, low pH, hyperuricosuria, or anatomical factors. Conversely, changes in one urinary analyte do not necessarily result in a symptomatic or radiologically progressive stone during short follow-up. Future studies should integrate both mechanistic and clinical endpoints.

### 4.7. Limitations

This study has several important limitations. First, its retrospective, single-center design and ethnically homogeneous cohort limit generalizability. Second, all participants received GLP-1-based therapy; without an untreated or active-comparator group, pre-treatment incidence-rate analysis, or self-controlled design, treatment-attributable risk cannot be estimated. Third, although all recurrent events occurred after treatment initiation, complete patient-level dates were unavailable for robust time-to-event analysis. Fourth, excluding 33 patients with less than 6 months of documented follow-up may have introduced selection bias, while the remaining observation periods were unequal and relatively short. Fifth, semaglutide and tirzepatide dose, titration, adherence, interruptions, cumulative exposure, and treatment changes were incompletely documented. Sixth, imaging was performed as part of routine care rather than according to a standardized surveillance protocol, creating potential detection bias; residual or persistent stones may also be difficult to distinguish from true new formation despite clinical adjudication. Seventh, the comparison group combined patients without prior stones and patients with a single pre-treatment episode, and a baseline stone-former-only analysis was not possible from the available aggregate dataset. Eighth, the timing of 24 h urine collection relative to treatment initiation and recurrence was not standardized, and complete urinary calcium, oxalate, citrate, uric acid, sodium, pH, and supersaturation data were unavailable. Finally, only 13 events occurred, producing wide confidence intervals, limiting adjusted modeling, and requiring that the allopurinol finding and all between-group comparisons be regarded as exploratory. Stone composition analysis was unavailable because infrared spectrophotometry was not accessible at our institution.

## 5. Conclusions

In this Romanian retrospective cohort, 13 of 126 patients (10.3%) had a clinically and radiologically confirmed recurrent urinary stone event after initiation of semaglutide or tirzepatide. No measured characteristic clearly differentiated patients with and without recurrence, but the small number of events and wide uncertainty preclude claims of no association. Allopurinol was more frequent among recurrent stone formers in an unadjusted analysis, most plausibly reflecting confounding by indication. These findings complement metabolic studies suggesting no major adverse change in 24 h urine lithogenic parameters during GLP-1-based weight loss, but they do not establish that GLP-1-based therapy increases, decreases, or does not alter recurrence risk. Prospective studies should enroll patients before treatment initiation, document baseline imaging and stone history, standardize imaging and 24 h urine timing, capture gastrointestinal adverse effects and detailed drug exposure, and use an untreated or active-comparator design with time-to-event analysis.

## Figures and Tables

**Table 1:** Baseline clinical, metabolic, renal, and treatment characteristics.

Characteristic	Overall Cohort, N = 126
Age, years	67.00 [62.25–70.00]
BMI, kg/m^2^	32.00 [30.25–34.00]
Weight change, kg	−15.00 [−17.25–−14.00]
HbA1c, %	6.90 [6.70–7.00]
24 h urine volume, mL	2200.00 [1990.00–2350.00]
Serum creatinine, mg/dL	1.00 [0.90–1.00]
eGFR, mL/min/1.73 m^2^	69.50 [60.00–77.00]
Follow-up, months	9.00 [8.00–12.00]
Female sex	59 (46.8%)
Smoking	42 (33.3%)
Dyslipidemia	112 (88.9%)
Diabetes mellitus	103 (81.7%)
Semaglutide	94 (74.6%)
Tirzepatide	32 (25.4%)
Recurrent urinary stone disease	13 (10.3%)
Serum sodium at GLP-1 initiation, mmol/L	139.00 [137.00–140.00]
Serum potassium at GLP-1 initiation, mmol/L	4.31 [4.12–4.65]

**Table 2:** Concomitant medications relevant to urinary stone risk.

Medication	Overall *n* (%)	No Recurrent Stones	Recurrent Stones	OR	95% CI Low	95% CI High	*p* Value
Metformin	80 (63.5%)	72/113 (63.7%)	8/13 (61.5%)	0.91	0.28	2.97	1.000
SGLT2 inhibitor	32 (25.4%)	30/113 (26.5%)	2/13 (15.4%)	0.50	0.11	2.40	0.513
Allopurinol	52 (41.3%)	43/113 (38.1%)	9/13 (69.2%)	3.66	1.06	12.63	0.039
Diuretic	31 (24.6%)	29/113 (25.7%)	2/13 (15.4%)	0.53	0.11	2.52	0.517

**Table 3:** Baseline serum sodium and potassium according to recurrent stone status.

Variable	Overall	No Recurrent Stones	Recurrent Stones	*p* Value
Sodium at initiation, mmol/L	139.00 [137.00–140.00]	139.00 [137.00–140.00]	140.00 [139.00–140.00]	0.186
Potassium at initiation, mmol/L	4.31 [4.12–4.65]	4.30 [4.12–4.63]	4.63 [4.06–4.85]	0.692

**Table 4:** Clinical characteristics according to recurrent urinary stone status.

Variable	No Recurrent Stones	Recurrent Stones	*p* Value
Age, years	67.00 [62.00–70.00]	67.00 [66.00–68.00]	0.460
BMI, kg/m^2^	32.00 [31.00–34.00]	33.00 [30.00–34.00]	0.715
Weight change, kg	−16.00 [−17.50–−14.00]	−14.50 [−16.00–−14.00]	0.442
HbA1c, %	6.90 [6.70–7.00]	6.80 [6.00–7.00]	0.578
24 h urine volume, mL	2200.00 [1967.50–2350.00]	2300.00 [1990.00–2350.00]	0.804
Serum creatinine, mg/dL	1.00 [0.90–1.00]	1.00 [1.00–1.00]	0.132
eGFR, mL/min/1.73 m^2^	70.00 [60.00–77.00]	64.00 [54.00–77.00]	0.379
Follow-up, months	9.00 [8.00–10.00]	12.00 [9.00–12.00]	0.151
Female sex	51/113 (45.1%)	8/13 (61.5%)	0.380
Smoking	38/113 (33.6%)	4/13 (30.8%)	1.000
Dyslipidemia	101/113 (89.4%)	11/13 (84.6%)	0.638
Diabetes mellitus	92/113 (81.4%)	11/13 (84.6%)	1.000
Semaglutide	83/113 (73.5%)	11/13 (84.6%)	0.513

## Data Availability

The de-identified data underlying this study may be made available by the corresponding author upon reasonable request, subject to institutional approval and applicable privacy regulations.

## Abbreviations

The following abbreviations are used in this manuscript:

GLP-1	glucagon-like peptide-1
GIP	glucose-dependent insulinotropic polypeptide
SGLT2	sodium–glucose cotransporter 2
BMI	body mass index
eGFR	estimated glomerular filtration rate
CT	computed tomography
IQR	interquartile range
OR	odds ratio
CI	confidence interval
